# Who Should Rather Undergo Transesophageal Echocardiography to Determine Stroke Etiology: Young or Elderly Stroke Patients?

**DOI:** 10.3389/fneur.2020.588151

**Published:** 2020-12-18

**Authors:** Christoph Strecker, Felix Günther, Andreas Harloff

**Affiliations:** ^1^Department of Neurology and Neurophysiology, Faculty of Medicine, Medical Center – University of Freiburg, Freiburg, Germany; ^2^Department of Internal Medicine, Staufenburg Klinik, Durbach, Germany

**Keywords:** transesophageal echocardiography, stroke, cerebral embolism, aortic atheroma, patent forame ovale

## Abstract

**Introduction:** The indication of transesophageal echocardiography (TEE) in acute stroke is unclear. Thus, we systematically studied the impact of TEE on determining stroke etiology and secondary prevention in patients of different age-groups with cryptogenic stroke.

**Methods:** Four hundred and eighty five consecutive patients with acute retinal or cerebral ischemia were prospectively included and underwent routine stroke workup including TEE. Stroke etiology was identified according to the TOAST classification and patients were divided in those with determined and cryptogenic stroke etiology without TEE results. Then, the frequency of high- and potential-risk sources in TEE was evaluated in <55, 55–74, and ≥75 year-old patients with cryptogenic stroke etiology.

**Results:** Without TEE, stroke etiology was cryptogenic in 329(67.8%) patients and TEE determined possible etiology in 158(48.4%) of them. In patients aged <55, 55–74, ≥75, TEE detected aortic arch plaques ≥4 mm thickness in 2(1.2%), 37(23.0%), and 33(40.2%) and plaques with superimposed thrombi in 0(0.0%), 5(3.1%), and 7(8.5%); left atrial appendage peak emptying flow velocity ≤30cm/s in 0(0.0%), 1(0.6%), and 2(2.4%), spontaneous echo contrast in 0(0.0%), 1(0.6%), and 6(7.3%), endocarditis in 0(0.0%), 0(0.0%), and 1(1.2%) and patent foramen ovale (PFO) plus atrial septum aneurysm (ASA) in 18(20.9%), 32(19.9%), and 14(17.1%), respectively. TEE changed secondary prevention in 16.4% of these patients following guidelines of 2010/11 and still 9.4% when applying the guidelines of 2020.

**Conclusions:** TEE was highly valuable for determining stroke etiology and influenced individual secondary prevention based on available treatment guidelines and expert opinion in most cases. In young patients the impact of TEE was limited to the detection of septal anomalies. By contrast, in older patients TEE detected high numbers of complex aortic atheroma and potential indicators of paroxysmal atrial fibrillation.

## Introduction

Transesophageal echocardiography (TEE) is the current gold standard for the detection of cardiac and aortic embolic sources (1, 2). However, both the reported frequencies of embolic sources and the benefit for therapeutic management are conflicting (3). A recent systematic review revealed a marked inter-study variation in terms of the prevalence of such findings in patients <55 and >55 years in TEE. Moreover, the initiation of oral anticoagulation following TEE ranged from 0 to 31% in patients with cryptogenic stroke (4). Currently, clear guidelines for TEE performance in acute stroke patients do not exist which is also due to the lack of evidence for the optimal treatment of many pathologies that are detected by TEE.

Cardiac and aortic findings in TEE differ according to patients age (4). In younger patients, patent foramen ovale (PFO) is the most common finding in TEE. Large trials have recently demonstrated the advantage of PFO-closure over conservative therapy (5, 6) and TEE, the reference method for the detection and characterization of PFO, was required prior to PFO-closure in these trials. With increasing age, both complex aortic plaques and indicators of paroxysmal atrial fibrillation, such as spontaneous echo contrast (SEC) or reduced left atrial appendage (LAA) peak emptying flow velocity or LAA-thrombi, become more prevalent (7). Especially, complex plaques of the descending aorta are frequent in the elderly and recent studies have emphasized their role as a potential and yet neglected source of brain embolization through reverse blood flow in diastole (8, 9).

Therefore, it was our aim to systematically investigate the impact of TEE for the determination of stroke etiology and treatment in patients in patients of different age with acute and cryptogenic stroke after routine diagnostics including the detection of complex plaques of the proximal descending aorta as an embolic source. Moreover, we evaluated the impact of TEE findings on individual secondary prevention according to the former guidelines of 2010/2011 (10, 11) and the current German S2e-guideline of 2020 (12).

## Materials and Methods

### Study Population

**Figure 1:**
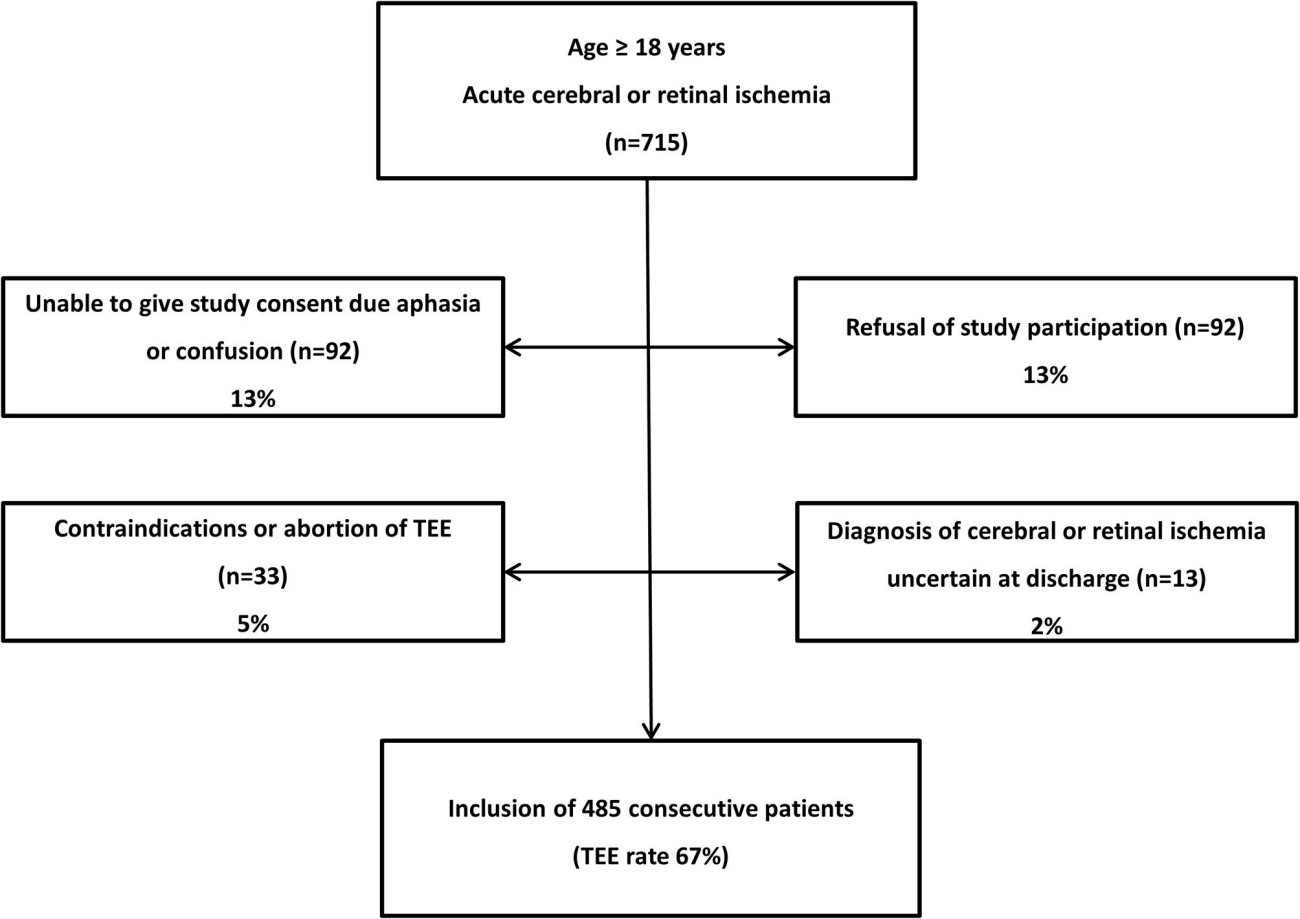
Flowchart of patients‘ screening and inclusion and exclusion of consecutive patients.

From August 2010 to April 2011, 715 consecutive patients admitted to our stroke unit fulfilled the inclusion criteria of ≥18 years of age and acute cerebral (stroke or transient ischemic attack) or retinal ischemia. In order to obtain optimal and representative information regarding the additional diagnostic impact of TEE every patient with acute cerebral or retinal ischemia was offered to participate in the study and to undergo TEE. Accordingly, TEE was also performed in patients with e.g., atrial fibrilliation or high-grade internal carotid artery stenosis for study reasons. Next to the standard insurance of our hospital we therefore contracted insurance to cover potential adverse events related to study TEE. Of the initial 715 patients, 485 were included in the final study cohort resulting in a TEE rate of 67.8% (Figure 1).

Patients' demographics and cardiovascular risk factors were obtained from patients' electronical charts and personal interviews. Written informed consent was obtained from all patients or their next-on-kin. The local ethics committee of our institution approved the study.

### Stroke Diagnostics

All patients received the following routine diagnostics: brain imaging with computed tomography (CT) in 140 (28.7%), magnetic resonance imaging (MRI) in 180 (37.1%) or both, in 165 (34.0%) patients, CT-angiography in 37 (7.6%), MR-angiography in 312 (64.3%). All patients received Duplex sonography of extracranial and intracranial arteries. Furthermore, 12-channel electrocardiography (ECG), 24-h Holter-ECG or 72-h ECG monitoring was executed during the stay on the stroke unit. Transthoracic and transesophageal echocardiography (TTE and TEE) were performed by two experienced cardiologists in all patients and within a median of 2 days after admission. Patients ≤60 years received additional screening for thrombophilia.

### Stroke Etiology

In a first step, stroke etiology was classified according to the Trial of Org 10172 in Acute Stroke Treatment (TOAST) criteria (13) without considering TEE data. In a second step, stroke etiology was reclassified after considering TEE data in patients with cryptogenic stroke. This was done to identify the additional information and therapeutic consequences arising from TEE findings.

High-risk embolic sources according to the TOAST classification derived from TEE were: endocarditis, thrombus in the left atrial cavity or appendage, complex aortic plaques including plaques of the descending aorta (DAo), i.e., the first 3 cm downstream of the outlet of the left subclavian artery, defined as ≥4 mm thickness and/or superimposed aortic thrombi. Potential risk sources were: septal anomalies (PFO, atrial septum aneurysm (ASA), atrial septal defect (ASD), PFO+ASA), spontaneous echo contrast (SEC), left atrial flow velocity ≤30 cm/s, and aortic plaques <4 mm including plaques of the proximal Dao (i.e., the first 3 cm downstream of the outlet of the left subclavian artery). TOAST classification was performed by one senior neurologist and by another senior neurologist for final decision in case of uncertainty.

Then, patients were distributed to three different age groups. Group 1 represented stroke patients aged <55 years. For a detailed evaluation of age dependent effects, patients ≥55 years were divided into two subgroups: group 2 containing those aged ≥55– <75 years, and group 3 those with an age ≥75 years.

### Therapeutic Management Following TEE Performance

In a last step the impact of TEE findings on secondary prevention was examined. Therapeutic management was based on the European and German stroke guidelines from 2008 which were valid between August 2010 and April 2011 (10, 11). In some cases the therapeutic management was based on the decision of the treating physician as off-label therapy, i.e., initiating oral anticoagulation in patients with aortic thrombi or SEC. However, current guidelines (12) no longer recommend oral anticoagulation for PFO and concomitant ASA. Instead, they recommend PFO-closure in patients ≤60 years of age with otherwise cryptogenic stroke etiology and visible and embolic infarction pattern on cerebral imaging (MRI or CT). Thus, we evaluated brain imaging of these PFO-patients with respect to a visible acute territorial or cortical infarction presuming an embolic pattern. Accordingly, we calculated the number of PFO-patients <60 years of age who would be candidates for PFO closure following current stroke guidelines in 2020.

### Transesophageal Echocardiography

All TEE examinations were performed by two experienced cardiologists using a Philips iU22 (Philips Healthcare, Best, The Netherlands) ultrasonic device and a 2–7 MHz ultrasound probe (S7-2t Omni Sector transesophageal phased array) based on the recommendations and standards of the American Society of Echocardiography (14) and as described previously (15). The left atrium was examined thoroughly with respect to SEC and thrombi. When a fixed or mobile echo-dense mass could be clearly differentiated from the wall of the left atrial cavity/LAA a thrombus was diagnosed. For measurement of LAA end-diastolic peak flow velocity, the Doppler sample volume was positioned in the proximal third of the appendage and the average taken of the values of five cycles. ASA was diagnosed when the maximum excursion of an abnormally redundant and mobile atrial septum was ≥10 mm. Injections of an agitated contrast agent were performed at rest and during Valsalva maneuver. An atrial right-to-left shunt was diagnosed when microbubbles were detected in the left atrium within four cardiac cycles after right atrial opacification. All aortic segments were examined with respect to aortic plaques defined as irregular intimal thickening with increased echogenicity. Maximum plaque thickness was measured manually and presence of mobile components/thrombi was recorded.

### Statistical Analysis

Data are presented as mean and standard deviation or median (interquartile range) for continuous variables and as absolute frequencies and percentages for categorical variables. Departures from normality were detected with the Shapiro-Wilk statistics. To detect statistically significant relations between categorical variables, Fisher's exact test was used. Depending on data distribution two-tailed *t* tests or non-parametric tests were applied as appropriate for continuous variables. A two-tailed *p*-value < 0.05 was considered to indicate statistical significance. All analyses were performed with IBM SPSS Statistics 22.

## Results

### Patients' Characteristics

Baseline characteristics of the 485 participants are shown in Table 1. Table 2 demonstrates the results of the TOAST classification (a) when considering routine diagnostics and *excluding* information from TEE and (b) when TEE results were *included* for classification in order to identify the effect of TEE information. After routine diagnostics, stroke etiology was cryptogenic in 329/485 (67.8%) patients. After incorporating TEE information, etiology was determined in another 158/485 patients (32.5%). In those, 24 (22.0%) patients were <55 years-old, 81 (32.6%) patients were ≥55– <75 years-old, and 53 (41.3%) patients were ≥75 years-old, respectively.

**Table 1:** Baseline characteristics of the 485 study participants.

**Characteristic**	**All patients *n* = 485**	**Determined etiology prior to TEE *n* = 156**	**Cryptogenic etiology prior to TEE *n* = 329**	***P*-value**
Age in years—mean ± SD	64.3 ± 14.5	67.0 ± 12.8	63.0 ± 15.0	0.020
Females, no (%)	209 (43.1%)	69 (44.2%)	140 (42.6%)	0.695
Hypertension, no (%)	341 (70.3%)	117 (75.0%)	224 (68.1%)	0.169
Hyperlipidemia, no (%)	132 (27.2%)	45 (28.8%)	87 (26.4%)	0.663
Diabetes, no (%)	120 (24.7%)	49 (31.4%)	71 (21.6%)	0.025
Smoking, no (%)	106 (21.9%)	36 (23.1%)	70 (21.3%)	0.725
Stroke/TIA, no (%)	78 (16.1%)	28 (17.9%)	50 (15.2%)	0.509
CHD, no (%)	87 (17.9%)	37 (23.7%)	50 (15.2%)	0.031
PAD, no (%)	31 (6.4%)	16 (10.3%)	15 (4.6%)	0.027
Atrial fibrillation, no (%)	55 (11.3%)	55 (35.3%)	0 (0.0)	<0.001
Left ventricular ejection fraction in %–median (IQR)	55 (55-60)	55 (55-60)	60 (55-60)	<0.001

*Stroke/TIA, history of cerebral ischemia; CHD, history of coronary heart disease; PAD, history of peripheral artery disease; ±SD, standard deviation; IQR, interquartile range*.

**Table 2:** Stroke etiology according TOAST criteria prior to and after considering TEE findings.

	**All patients** ***n*** **=** **485**		** <55 years** ***n*** **=** **109**		**≥55– <75 years** ***n*** **=** **248**		**≥75 years** ***n*** **=** **128**	
**TOAST classification, no (%)**	**– TEE data**	**+ TEE data**	**– TEE data**	**+ TEE data**	**– TEE data**	**+ TEE data**	**– TEE data**	**+ TEE data**
Large-artery atherosclerosis, no (%)	53 (10.9%)	131 (27.0%)	0 (0.0%)	6 (5.5%)	37 (14.9%)	83 (33.5%)	10 (7.8%)	42 (32.8%)
Presumed cardioembolism, no (%)	64 (13.2%)	116 (23.9%)	6 (5.5%)	29 (26.6%)	34 (13.7%)	52 (21.0%)	24 (18.8%)	35 (27.3%)
Small-vessel disease, no (%)	21 (4.3%)	13 (2.7%)	1 (0.9%)	1 (0.9%)	11 (4.4%)	5 (2.0%)	9 (7.0%)	7 (5.5%)
Other etiology, no (%)	15 (3.1%)	12 (2.5%)	10 (9.2%)	8 (7.3%)	4 (1.6%)	4 (1.6%)	1 (0.8%)	0 (0.0%)
≥1 probable etiology, no (%)	3 (0.6%)	42 (8.7%)	0 (0.0%)	3 (2.8%)	1 (0.4%)	24 (9.7%)	2 (1.6%)	15 (11.7%)
Cryptogenic etiology, no (%)	329 (67.8%)	171 (35.3%)	86 (78.9%)	62 (56.9%)	161 (64.9%)	80 (32.3%)	82 (64.0%)	29 (22.7%)a

*Other etiology, other determined etiology*.

### Findings in Patients With Determined Stroke Etiology Prior to TEE

Without TEE information 64/485 (13.2%) patients were classified as stroke of cardioembolic etiology according to the TOAST criteria. Cardioembolic sources detected without TEE were: paroxysmal atrial fibrillation in 37 (57.8%), persistent atrial fibrillation in 18 (28.1%), sick sinus syndrome in 1 (1.6%), left ventricular aneurysm in 3 (4.7%), recent myocardial infarction <4 weeks in 1 (1.6%), mechanical heart valve in 2 (3.1%) and akinetic left ventricular segment in 7 (10.9%) patients in this group. Without consideration of TEE information stroke etiology was classified as large-artery atherosclerosis in 53/485 (10.9%) patients. Thirty-eight (71.7%) of them had a high-grade stenosis or an occlusion of the internal carotid artery ipsilateral to the side of stroke, 7 (13.2%) had a high-grade stenosis of the extracranial vertebral artery and a corresponding brain stem infarction, and 8 (15.1%) patients showed an intracranial stenosis. Furthermore, 15/485 (3.1%) patients were classified as other etiology according to the TOAST criteria. Of these, 5 (33.3%) showed an extracranial artery dissection, 3 (20.0%) had a known antiphospholipid syndrome, 3 (20.0%) a CADASIL-syndrome, 1 (6.6%) a giant cell arteritis, 2 (13.3%) a cerebral vasculitis and 1 (6.6%) had a venous infarction as a result of a septic sinus thrombosis.

### TEE Findings in Patients With Cryptogenic Stroke Etiology

Table 3 shows the cardiac and aortic findings after incorporating TEE information.

**Table 3:** TEE-findings in all 485 patients, in all patients with cryptogenic etiology and with cryptogenic etiology in different age groups.

	**All patients *n* = 485**	**Cryptogenic etiology *n* = 329**	** <55 years *n* = 86**	**≥55 and <75 years *n* = 161**	**≥75 years *n* = 82**
Endocarditis, no (%)	3 (0.6%)	1 (0.3%)	0 (0.0%)	0 (0.0%)	1 (1.2%)
SEC, no (%)	34 (7.0%)	7 (2.1%)	0 (0.0%)	1 (0.6%)	6 (7.3%)
Vmax LAA ≤30cm/s, no (%)	19 (3.9%)	3 (0.9%)	0 (0.0%)	1 (0.6%)	2 (2.4%)
LA/LAA-thrombus, no (%)	3 (0.6%)	0 (0.0%)	0 (0.0%)	0 (0.0%)	0 (0.0%)
PFO, no (%)	44 (9.1%)	34 (10.3%)	21 (24.4%)	10 (6.2%)	3 (3.7%)
ASA, no (%)	23 (4.7%)	17 (5.2%)	2 (2.3%)	4 (2.5%)	11 (13.4%)
PFO plus ASA, no (%)	91 (18.8%)	64 (19.5%)	18 (20.9%)	32 (19.9%)	14 (17.1%)
ASD, no (%)	6 (1.2%)	5 (1.5%)	4 (4.7%)	1 (0.6%)	0 (0.0%)
Aortic plaque <4 mm, no (%)	316 (65.2%)	204 (62.0%)	13 (15.1%)	120 (74.5%)	71 (86.6%)
Aortic Arch plaque ≥4 mm, no (%)	113 (23.1%)	72 (21.6%)	2 (1.2%),	37 (23.0%)	33 (40.2%)
Aorta descendens plaque ≥4 mm, no (%)	197 (40.6%)	126 (38.1%)	4 (3.5%)	72 (44.7%)	50 (60.1%)
Aortic thrombus, no (%)	16 (3.3%)	12 (3.6%)	0 (0.0%)	5 (3.1%)	7 (8.5%)

*Please note that all aortic plaques shown here include plaques of the proximal descending aorta. SEC, spontaneous echo contrast; Vmax-LAA, maximal left atrial appendage velocity; LAA/LA, left atrial appendage/left atrium; PFO, patent foramen ovale; ASA, atrial septum aneurysm; ASD, atrial septum defect*.

The three main findings in patients with cryptogenic stroke (please see Table 3 for details) were:

- In patients <55 years, isolated PFO (24.4%) and PFO with ASA (20.9%) were the by far most prevalent findings. In none of the patients we observed indicators of paroxysmal atrial fibrillation nor aortic plaques with superimposed thrombi in TEE.
- In patients ≥55 and <75 years, the prevalence of isolated PFO was 6.2%, and of PFO plus ASA 19.9%. However, TEE detected SEC in one patient (0.6%) who also had LAAV ≤30 cm/s. Aortic thrombi were found in 3.7%, aortic plaques ≥4 mm in 49.1%.
- In patients ≥75 years, isolated PFO was found in 3.7% and PFO plus ASA in 17.1%. However, TEE revealed by far most cardiac and aortic high-risk sources in comparison to the other groups (for more details please see Table 3).

A thrombus in the left atrium or left atrial appendage was found in none of the patients with cryptogenic stroke. All three patients (0.6%) with LA/LAA thrombus in TEE had known atrial fibrillation.

### Therapeutic Management Following TEE Performance

**Figure 2:**
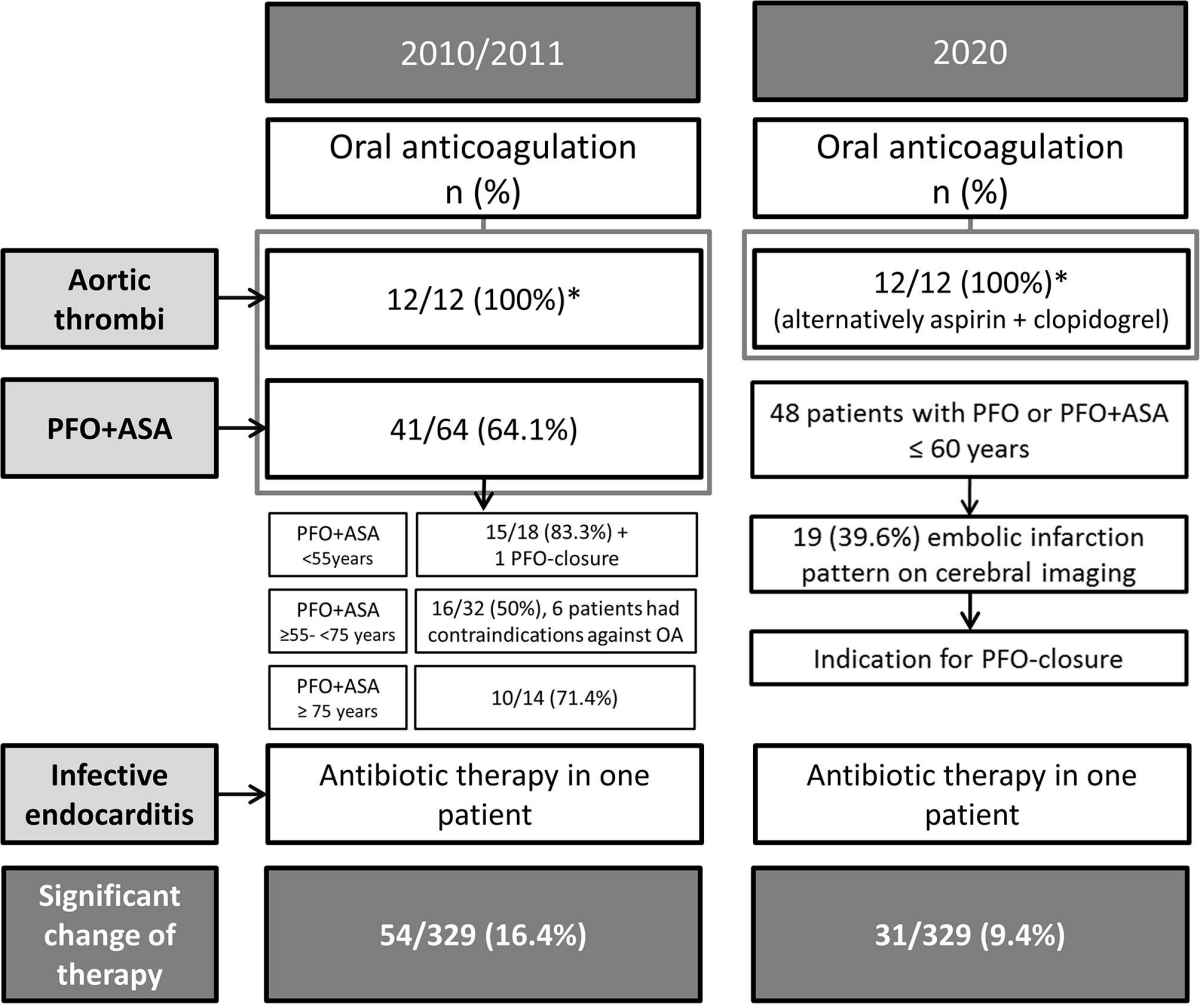
Therapeutic management based on TEE results in 2010/2011 **(left)** and transferred to current guidelines valid 2020 **(right)**. *Indicates off-label therapy based on the decision of the treating physician in 2010/2011, assuming that 2020 the decision is probably the same.

Additional TEE findings led to a change in therapeutic management in 16.4% (54/329) patients with previously cryptogenic stroke after routine diagnostics according to the guidelines that were valid in 2010/2011 (Figure 2). Isolated PFO or PFO plus ASA were found in 48 patients ≤60 years of age and with cryptogenic stroke. Nineteen of them (39.6%) showed an acute and embolic brain infarction pattern on cerebral imaging. Considering the current stroke guidelines they were candidates for PFO-closure nowadays. Thus, even taking current stroke guidelines into account TEE led to a significant change of individual secondary prevention (i.e., oral anticoagulation or PFO closure instead of platelet inhibibition etc.) in 9.4% (31/329) of our patients (Figure 2).

## Discussion

In this study, we prospectively investigated a large and consecutive cohort of patients with acute cerebral or retinal ischemia of cryptogenic etiology. In addition, we considered the very frequent but so far neglected complex plaques of the proximal descending aorta as a source of cerebral embolism. Finally, we provide representative data on the impact of TEE regarding the identification of possible stroke etiology and treatment in different age-groups.

TEE allowed to determine stroke etiology in almost half of the patients with previously cryptogenic stroke after routine diagnostics. Moreover, TEE resulted in a significant change of therapeutic management (i.e., oral anticoagulation or PFO closure instead of platelet inhibition etc.) in 16.4% of these patients based on the guidelines in 2010/2011. The main difference of the updated guidelines (12) is the recommendation to perform PFO closure instead of platelet inhibition or oral anticoagulation under the following conditions: in patients <60 years with cryptogenic stroke, evidence of PFO or PFO with ASA and embolic brain infarction.

In patients ≤55 years, the advantage of TEE was limited to the detection of septal anomalies. In patients ≥75 years, however, TEE contributed the most to clarify possible stroke etiology in up to 40%, especially due to the detection of complex aortic plaques and spontaneous echo contrast as an indicator of potential or previously unknown paroxysmal atrial fibrillation.

### Aortic Atheroma

Aortic plaques ≥4 mm including plaque thrombi are an independent predictor of recurrent stroke (16) but their optimal treatment is still unclear. The only trial in this regard, the Aortic arch Related Cerebral Hazard (ARCH) trial, compared aspirin plus clopidogrel vs. warfarin and found no superiority of either treatment. However, this trial was underpowered (17). To date, patients with aortic plaques ≥4 mm are treated with platelet inhibitors and statins. Patients with superimposed thrombi usually receive oral anticoagulation off-label for several weeks and are then switched to platelet inhibition (15, 18). Alternatively, they receive aspirin plus clopidogrel off-label and statins for 4–6 weeks and are then switched to aspirin or clopidogrel and statins which is supported by the results of the POINT trial (19). The number of patients with complex aortic plaques and cryptogenic stroke in our cohort (42%) was comparable to previous studies in cryptogenic stroke (20). Similar to these studies, most complex plaques were detected in the proximal descending aorta. A number of recent studies demonstrated the connection of complex atheroma located in the first 2–3 cm of the descending aorta with the brain by diastolic flow reversal. Therefore, they were considered in our present study and should also be considered as a potential source of cerebral embolism in clinical routine (8, 9). TEE is currently the reference method for the detection of complex aortic plaques. Harloff et al. (21, 22) reported high negative predictive values for the exclusion of complex aortic plaques and aortic thrombi in stroke patients with a normal carotid intima-media thickness (≤0.9 mm). Similarly, Barazangi et al. (23) reported high negative predictive values for computed tomography angiography in the detection of aortic arch atheroma, especially for high-grade atheroma. Accordingly, the application of such non-invasive diagnostic strategy as a screening tool could make TEE dispensable for the detection of complex aortic atheroma in individual cases with normal carotid arteries.

### Septal Anomalies

Interestingly, the prevalence of PFO plus ASA was comparable in patients <55 and ≥75 years which is in line with previous studies (24) whereas isolated PFO was ~6x more frequent in younger patients. PFO plus ASA led to one PFO-closure and oral anticoagulation in 64% of patients with cryptogenic stroke at the time of this study while isolated PFO was treated with antiplatelets based on former guidelines (10, 11). Recently published trials (5, 6) demonstrated the advantage for PFO-closure compared to treatment with antiplatelets or warfarin in patients <60 years with cryptogenic stroke and PFO. Thereby, the therapeutic impact of TEE has shifted: the number of patients receiving oral anticoagulation due to PFO in our analysis would decrease to a minimum nowadays. On the other hand, the apparent loss of a benefit of TEE would be countervailed by the PFO-closure that will be performed due to the detection by TEE in the majority of these patients instead. Moreover, TEE is required for the proper diagnosis of PFO before intervention. Considering the current guidelines, TEE would result in PFO closure in 40% of the patients of this cohort under the age of 60 years with PFO or PFO plus ASA. A number of studies (25–27) used transcranial Doppler ultrasound (“bubble-test”) or TTE to detect PFO non-invasively. Transcranial Doppler ultrasound is highly sensitive and specific for the detection of a cardiac right-to-left shunt even in patients with negative TTE or TEE (26, 27). Thus, if TEE is only indicated for the search of PFO (i.e., in stroke patients ≤55 years without a suspicion of aortic atheroma, cardiac disease or endocarditis) it is dispensable in case of a negative “bubble test” or a negative TTE.

### Indicators of Potential Atrial Fibrillation

SEC represents local blood stasis in the left atrial appendage and reduced LAA peak emptying flow velocity represents insufficient LAA myocardial function. Both are closely related to atrial fibrillation and thromboembolism (28). Similar to a recent meta-analysis (29) SEC and reduced LAA-velocity were rare in our patients with cryptogenic stroke who had not known atrial fibrillation. Compatible with that finding, no thrombus was found in the LA/LAA in this group. Current guidelines do not recommend oral anticoagulation when SEC or reduced LAA-flow are detected by TEE. While some authors doubt that SEC is clinically relevant in older patients at all (7), others report that SEC is associated with both a significantly higher risk of stroke or other embolic events and a reduced survival. Accordingly, patients with SEC and cryptogenic stroke may represent a subgroup in whom anticoagulation may be favorable (30). In our study, 4/7 patients with SEC and cryptogenic stroke were treated off-label with oral anticoagulation based on the decision of the treating physician. These four patients were older than 75 years and showed an embolic infarction on cerebral imaging, making paroxysmal atrial fibrillation as the underlying pathology highly probable. Due to the heterogeneous literature regarding the associated embolic risk of SEC that also depends on the grading of the SEC (31, 32) we did not attribute these four patients to a change in therapeutic management based on TEE. However, the detection of SEC or LA/LAA flow by TEE in such patients may be a valuable additional cardiac parameter indicating a yet undetected paroxysmal atrial fibrillation and should lead to an intensified search for paroxysmal atrial fibrillation that potentially includes the implantation of a reveal recorder. This is also supported by the fact that ongoing studies in patients with embolic stroke of undetermined stroke (ESUS), such as the ATTICUS-trial, have implemented these potential indicators for paroxysmal atrial fibrillation as risk factors for cerebral embolism in their inclusion criteria (33).

### Considerations for a Diagnostic Workflow in Future Stroke Patients

TEE is the gold standard for the detection of cardiac and aortic embolic sources but it is semi-invasive, requires sedation and/or local pharyngeal anesthesia and patients frequently feel discomfort or fear and reject the examination. Recommendations for TEE in stroke patients are heterogeneous. Warner et al. (34) did not recommend TEE for routine use because patients in sinus rhythm usually had findings in TEE resulting in a treatment with aspirin. In contrast, Strandberg et al. (35) and Harloff et al. (15) proposed that TEE should be performed in all stroke patients with cryptogenic stroke after routine diagnostics and without contraindications against oral anticoagulation. Vitebskiy et al. (7) concluded that TEE appears to be unwarranted in older patients despite the high incidence of cardiac and aortic findings, but lack of established and evidence based therapies for these conditions. Based on our data and considering the cited literature we propose the following approach for TEE indication in patients with acute cryptogenic stroke: The only finding resulting in oral anticoagulation in patients <55 years of our cohort was PFO plus ASA. Nowadays, PFO detection plus/without ASA in cryptogenic and embolic stroke would result in PFO closure in patients <60 years of age. However, TEE may be dispensable if a right-to-left shunt is excluded by TCD or TTE and if there are no signs of endocarditis. TEE is required in case of PFO detection in TCD in order to assess septal anatomy prior to PFO occlusion (5, 6). In patients ≥55 years we found a high rate of PFO and ASA but also aortic thrombi and SEC and reduced LAA-flow as indicators of paroxysmal atrial fibrillation. Thus, TEE should be considered in these patients if stroke etiology is cryptogenic after routine diagnostics and if there is an embolic infarction pattern on cerebral imaging. This applies especially for patients ≥75 years as this group showed the highest rate of mobile aortic thrombi and potential indicators of paroxysmal atrial fibrillation. Finally, in patients ≥55 years but ≤60 years TEE may be dispensable if there is no evidence for a right to left shunt in TCD or TTE and a normal carotid intima-media thickness <0.9 mm making aortic thrombi highly unlikely (21, 22).

### Limitations

The rate of TEE with regard to all admitted stroke patients was ~70%, indicating a minor selection bias because many patients with known etiology (such as atrial fibrillation or symptomatic high-grade internal carotid artery stenosis) refused to undergo additional TEE for research purposes. However, this TEE rate is by far higher than that in clinical routine in most stroke centers and significantly higher than the recommended TEE rates demanded by the official certification criteria for a German stroke unit recommending a minimal rate of TEE of 15% (36). Thus, we believe that this bias is of minor importance considering the large cohort and the detailed stroke workup. Especially, the patient group with most therapeutic consequences following TEE examination, i.e., patients with cryptogenic stroke, was high and thus representative. A systematic grading of the extent of SEC (31, 32) would be beneficial in future studies to better identify patients who are at high risk for thromboembolic events and require an intensified cardiac monitoring for atrial fibrillation and ultimately oral anticoagulation. It would be interesting to know how many patients with/without SEC or reduced LAA showed AF over the course of time. Such follow-up could improve the determination of stroke etiology and the assessment of the therapeutic benefit of TEE. Furthermore this could solve another general problem, namely that for many TEE findings there are currently no evidence-based treatment recommendations. Thus, many therapeutic decisions based on TEE results, i.e., initiating an oral anticoagulation in aortic thrombi, are rather based on expert opinion than on robust evidence as long as results from randomized-controlled trials are missing. Some of the therapeutic recommendations of 2010/2011, i.e., oral anticoagulation in stroke patients with PFO and ASA, are no longer valid. Instead, PFO occlusion has replaced oral anticoagulation in some of these patients based on the updated guidelines. Thus, we also provide updated therapeutic recommendation considering the current stroke guidelines. We believe that our results are still highly valid today since stroke diagnostics including TEE have not significantly changed in methodology or application algorithms in the past years.

## Conclusion

TEE contributed to the determination of individual possible stroke etiology in ~50% of our patients with previously cryptogenic stroke etiology after routine diagnostics. Based on available guidelines but especially based on expert opinion, TEE led to a significant change in secondary prevention in 16.4% of these patients and still in 9.4% of the patients when applying treatment guidelines of 2020. However, evidence-based guidelines for the treatment of many TEE findings are still lacking, except PFO-closure in stroke patients ≤60 years and treatment of endocarditis by antibiotics or cardiac valve repair. The only benefit of TEE in patients <55 years was the detection of septal anomalies. Thus, TEE may have been dispensable for diagnostic purposes in many of these younger patients in case of a negative TTE or TCD. Older patients ≥75 years showed the greatest impact regarding clarifying possible stroke etiology, mainly due to detection of complex aortic plaques and aortic thrombi and indicators of paroxysmal atrial fibrillation and also due to a high prevalence of PFO plus ASA. The optimal treatment of many pathologies detected by TEE in the elderly, however, is still unclear and requires further investigation by future randomized-controlled trials which are, therefore, urgently needed.

## Data Availability Statement

The raw data supporting the conclusions of this article will be made available by the authors, without undue reservation.

## Ethics Statement

The studies involving human participants were reviewed and approved by Ethics commitee of the Albert-Ludwigs University of Freiburg, Germany. The patients/participants provided their written informed consent to participate in this study.

## Author Contributions

CS performed the data analysis and interpretation and drafted the manuscript. FG carried out the TEE examinations and evaluations, supplemented parts of the manuscript, and revised it critically. AH drafted the study concept, conducted parts of the data analysis and revised, and supplemented the manuscript substantially. All authors contributed to the article and approved the submitted version.

## Conflict of Interest

The authors declare that the research was conducted in the absence of any commercial or financial relationships that could be construed as a potential conflict of interest.
